# Impacts of artificial light on food intake in invasive toads

**DOI:** 10.1038/s41598-020-63503-9

**Published:** 2020-04-16

**Authors:** Hirotaka Komine, Shinsuke Koike, Lin Schwarzkopf

**Affiliations:** 1 Institute of Global Innovation Research, Tokyo University of Agriculture and Technology, 3-5-8, Saiwai-cho, Fuchu, Tokyo 183-8509 Japan; 2 College of Science and Engineering, Centre for Biodiversity & Climate Change, James Cook University, Townsville, 4811 Australia

**Keywords:** Invasive species, Ecology, Urban ecology

## Abstract

Artificial light at night (ALAN) is a major form of anthropogenic disturbance. ALAN attracts nocturnal invertebrates, which are a food source for nocturnal predators, including invasive species. Few studies quantify the effects of increased food availablity by ALAN on invasive vertebrate predators, and enhancement of food intake caused by ALAN may also be influenced by various environmental factors, such as proximitity to cities, moon phase, temperature, rainfall and wind speed. Revealing the potential impacts on invasive predators of ALAN-attracted invertebrates, and the influence of other factors on these effects, could provide important insights for the management of these predators. We constructed and supplied with artificial light field enclosures for invasive toads, and placed them at locations with different levels of ambient light pollution, in northeastern Australia. In addition, we determined the effect of rainfall, temperature, wind speed, and lunar phase on food intake in toads. We found that ALAN greatly increased the mass of gut contents of invasive toads compared to controls, but that the effect was increased in dark lunar phases, and when there were low ambient light pollution levels. Effects of rainfall, temperature and wind speed on food intake were comparatively weak. To avoid providing food resources to toads, management of ALAN in rural areas, and during dark lunar phases may be advisable. On the contrary, to effectively capture toads, trapping using lights as lures at such times and places should be more successful.

## Introduction

Human-caused degradation of the natural environment is increasing, and a major driver of such degradation is artificial light at night (ALAN)^1,2^. Over the last 100 years, since lighting technologies were developed and urbanization has progressed^3^, ALAN has disrupted the natural nocturnal environment, worldwide. Presently, 23% of the earth’s land mass experiences ALAN^4^, and the level of light pollution is growing approximately 2% a year^5^. Because most terrestrial biota have been exposed to regular cycles of sunlight and darkness throughout evolutionary history, disruption of the nocturnal environment by ALAN has ecological impacts on the biota^6^.

In just a few decades, there has been a broad range of studies on the impacts of ALAN. For example, ALAN can alter foraging behaviour^7–9^, migratory behaviour^10,11^, physiology^12,13^ and mortality rates^14^. More recently, some studies have found that population dynamics^15^ and species interactions^16^ can also be altered by ALAN. Whereas it is clear that ALAN can have significant effects on wildlife, its impacts may be complex. For example, many kinds of nocturnal invertebrates, such as beetles, flies, and moths, are attracted to artificial light ^17,18^, which may have significant implications for populations and communities of these groups^19,20^. In addition, however, attracted invertebrates are a food source for nocturnal predators of invertebrates, such as geckos, anurans, bats, and birds^21–23^. Although such phenomena are well documented^21,23^, few studies quantify the effect of increased food availablity on predators, although they may be among the strongest impacts of ALAN. If, for example, mesopredators are attracted to areas with ALAN, and then consume other groups, this could be an important impact on community dynamics, given the impact of mesopredator release in other systems^24^.

Furthermore, the effect of increased food availability on predators of invertebrates caused by ALAN may be influenced by various environmental factors, because the amount of invertebrates attracted to ALAN probably varies with weather and environment. For example, increasing temperature or rainfall may increase the number of invertebrates attracted to ALAN^1,6^. On the other hand, increasing wind speed and ambient light, such as moon light, may decrease the effect of artificial light on invertebrate activity^1,6^. It is likely that these effects on invertebrate activity flow on to influence predation success. This chain of reasoning has not been examined, but to better predict the impacts of ALAN on predators of nocturnal invertebrates, it appears we may need to understand the influence of a variety of environmental variables on predation success.

Although many previous studies have examined the effects of ALAN on native species, few have examined interactions between ALAN and invasive species^25^. While both ALAN and invasive species are global issues causing biodiversity loss and degradation of ecosystem function, these issues have been considered separately^25^. They may interact, however, because invasive species often proliferate in urban areas, which are a major source of ALAN. There are many reasons why invasive species inhabit disturbed environments^26^, and ALAN may be a contributing factor. For example, the abundance of invasive house geckos was higher in artificially lit environments^27^, and they may be more willing to use artificially lit environments to obtain food resources than are native geckos, suggesting ALAN might contribute to their invasion success, globally^28^. Revealing the potential impact on invasive species of ALAN could provide important insights for the management of invasive species.

Cane toads (*Rhinella marina*) are a tropical invasive species, originally native to south America^29,30^. Originally introduced to control agricultural pests^31^, they have been introduced to the Carribean, most Pacific Islands, several Japanese Islands, and to Papua New Guinea and Australia. Famously, cane toads produce highly toxic secretions stored in their parotoid glands, which are used as anti-predator defences^30^. It has been well documented in Australia that when native predators, including snakes, lizards, crocodiles, and marsupials attempt to consume toads, they are often poisoned^31–36^. Thus toads typically have negative impacts on the native biota, and require management globally. Toads feed on nocturnal invertebrates, and ALAN provides an artificially large food resource for toads^37,38^. Although ALAN may have a positive effect on the invasion success and proliferation of toads, environmental factors influencing the food intake of toads have not been investigated. Revealing environmental factors associated with the indirect effects of ALAN on toads could contribute to efficient management of invasive cane toads.

Here, we experimentally quantified the influence of ALAN on food intake in toads. In addition, we determined the effect of four environmental factors: ambient light, temperature, rainfall and wind speed on food intake by toads in the field. We constructed field enclosures supplied with artificial light for toads in northeast Australia. The toads were allowed to feed freely overnight, after which they were euthanised, and we measured the mass and taxonomic composition of their gut contents, and categorized them as ‘flying’ or ‘non-flying’. We hypothesized that the mass of the gut contents of toads would increase with increasing temperature and rainfall, and with decreasing wind speed. We based these predictions on the likely impact of these environmental variables on invertebrate activity^1,6^. Similarly, we hypothesized that the mass of toad gut contents would increase with decreasing lunar phase, and with ambient light pollution levels. We based these predictions on the likely impact of light sources (i.e., the moon and ALAN) on invertebrate activity in the vicinity of our artificial light, and assumed that toads would eat more if more invertebrates were available.

## Material and methods

### Experimental design

**Figure 1:**
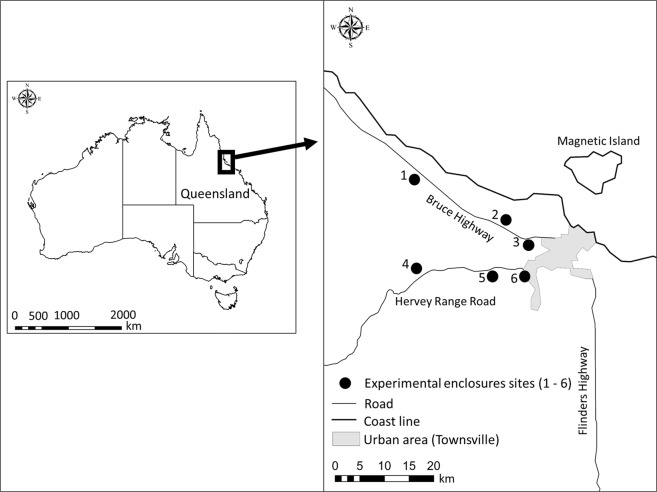
A map of study area. The urban area (Townsville, Queensland, Australia) is shown in grey. The 6 experimental enclosure sites are shown as black circles. Roads are shown as light lines, and coastlines as heavier lines.

**Figure 2:**
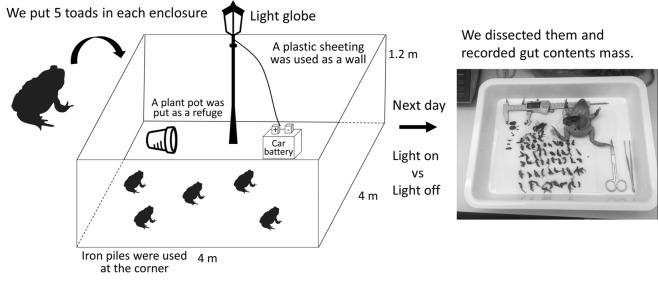
Overview of the enclosure experiment. We constructed six outdoor experimental enclosures in open eucalypt forest, and put 5 toads in each enclosure. The following day, we dissected them and recorded the mass of prey in each of their guts.

We constructed six outdoor experimental enclosures in rural areas vegetated with open eucalypt forest (mainly popular gum [*Eucalyptus platyphylla*]) with a grassy understorey around Townsville, Queensland, Australia (Fig. 1). Three enclosures were set along Hervey’s Range road and the others were set along the Bruce Highway. The distance between these sites were 5–15 km (Fig. 1). We drove four iron piles [star pickets] into the ground at the corners and wrapped these with UV stable plastic sheeting as walls (Fig. 2, Supplementary Fig. S1). Each enclosure measured 4 * 4 m, with walls 1.2 m high, and we provided a plant pot as a refuge. Light globes (100 W, Fluorescent Light Bulb (A Type) EFA25ED/21-A101H, Asahi Electric CO., LTD. Osaka, Japan.) were placed 1.5 m high at the centre of each enclosure, and car batteries (which lasted all night without recharge) were used as a power supply for the lights (Fig. 2, Supplementary Fig. S1). This light bulb has three peaks in the spectrum (blue [400–450 nm], green [500–600 nm] and red [600–650 nm]) to simulate daylight. We collected toads from Townsville and kept them without food for 40 h to ensure they had empty guts. We provided toads with water before each trial, to ensure they were fully hydrated. We then added five, randomly selected toads to each enclosure before sunset, and toads were allowed to feed overnight within the enclosure. The next morning, all of them were humanely euthanised using an overdose of buffered MS-222 (tricaine methanesulfonate) in a bath, and dissected in the laboratory at James Cook University. We recorded the mass of prey from each toad’s gut, and calculated the average gut content mass per trial (per night and per enclosure). Because toads in a trial were non-independent, we used average gut content mass per trial, as our measure of ‘predation success’. We conducted each trial for six days with different toads each day (applying a light-on treatment and light-off control, alternately) at each enclosure from December 2017 to April 2018. We also recorded light pollution levels at each enclosure, once, at the dark-moon phase, using a light meter (LUX METER FT3424, HIOKI E.E. CORPORATION, Nagano, Japan). Light pollution levels were as follows: site 1: 0.00 Lx, site 2: 0.01 Lx, site 3: 0.22 Lx, site 4: 0.00 Lx, site 5: 0.01 Lx, site 6: 3.65 Lx. We categorized them as follows: 0.00 Lx: no light pollution, 0.01 Lx: low levels of light pollution, 0.22 Lx: moderate levels of light pollution, 3.65 Lx: high levels of light pollution. When the lights were on, light intensities on the floor of the enclosures were as follows: site 1: 7.34 Lx, site 2: 7.59 Lx, site 3: 7.57 Lx, site 4: 7.30 Lx, site 5: 7.81 Lx, site 6: 7.73 Lx.

The size of individual toads that went into the enclosures varied, and we thought it possible that toad body size may influence the amount they consumed. We tested for a relationship between gut content mass per individual and snout-vent length (SVL) using a linear regression, and there was no significant relationship between these variables, so toad body size is not reported further (p > 0.05 Supplementary Fig. S2. Supplementary Table S1).

Although we did not measure the influx of insects to the light, we assumed that the number of insects falling to the ground and available to toads was a measure of this variable. This seems a reasonable assumption because insect researchers often evaluate the abundance of insects using captured fallen insects attracted to light traps^39^. They also use the number of fallen insects attracted to light to forecast pest outbreaks for agriculture^40^.

We used available public weather data from the Australian Government Bureau of Meteorology to obtain temperature, rainfall and wind speed at each site^41^, and lunar phase from ‘timeanddate.com’^42^, which we used as covariates, potentially influencing mean toad gut content mass.

### Statistical analysis

To examine the relationships between mean mass of toad gut contents in the experimental enclosures, and various environmental factors, we used the ‘lme4’ package to conduct generalized linear mixed modelling (GLMM). The error distribution was identified as a Gaussian distribution. We used average gut content mass of all the toads in a trial as the response variable, and we used the experimental artificial light treatment (light-on vs light-off), light pollution level, temperature, rain fall, wind speed and lunar phase as explanatory variables. We also examined interactions between the experimental artificial light treatment and the light pollution level, the experimental artificial light treatment and lunar phase, and the light pollution level and lunar phase, to look for interactions between the impact of artificial light, and natural light. Enclosure number was included as a random effect. We standardized numeric explanatory variables (we subtracted the mean from each data point and divided by the standard deviation to scale the data to a mean of 0 and a variance of 1). We conducted model comparison using the ‘dredge’ function in the ‘MuMIn’ package to evaluate the relative importance of each explanatory variable. Additionally we tested whether percentage of flying invertabrates in gut contents of toads increased when lights were on using Welch Two Sample t-test. All statistical analysis was performed in R V 3.6.0^43^.

## Results

### Experimental trials

We conducted 37 trials (site 1, 2, 3, 5 & 6: 6 trials, site 4: 7 trials) (Supplementary Table S2) and we collected gut contents from 171 toads (site 1: 29 toads, site 2: 28 toads, site 3: 28 toads, site 4: 32 toads, site 5: 27 toads, site 6: 27 toads).

### Variables influencing mean food intake per experimental trial

**Figure 3:**
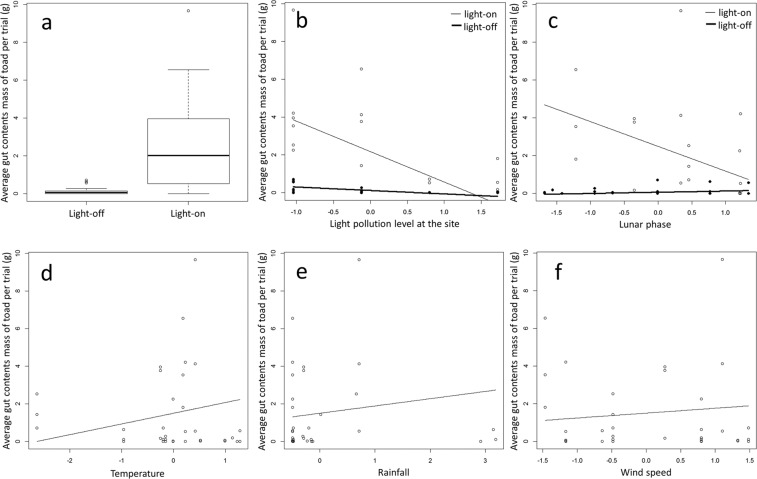
(**a**) Relationship between average toad gut content masses in experimental enclosures, and experimental light treatment (comparing all periods when the light was on versus all periods when the light was off). (**b**) The influence of ambient light pollution on average toad gut content masses in experimental enclosures when an artificial lights were on, and during dark controls. Gut contents masses when artificial lights were on are shown as white circles and a light line, and light-off treatment as black circles and heavier line. (**c**) The influence of lunar phase on average toad gut content masses in experimental enclosures when an artificial lights were on, and during dark controls. Mean gut content masses collected when lights were on are shown as white circles, and a light line, whereas mean gut content masses during light-off controls are shown as black circles and heavier line. (**d**) The influence of temperature on average toad gut content masses in experimental enclosures. (**e**) The influence of rainfall on average toad gut content masses in experimental enclosures. (**f**) The influence of wind speed on average toad gut content masses in experimental enclosures.

**Figure 4:**
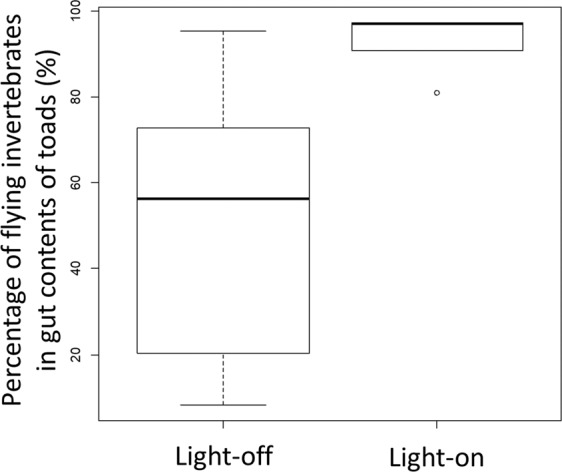
Percentage of flying invertebrates in gut contents of toads when lights were on and off.

Model comparisons showed that the experimental artificial light treatment (light-on vs light-off), ambient light pollution levels, lunar phase, and the interactions between experimental artificial light treatment and ambient light pollution level, and between the experimental artificial light treatment and lunar phase were selected in all models with delta AIC < 2 (Table 1). The light-on treatment increased mean gut content mass of invasive toads (Fig. 3a), but the impact of the experimental artificial light treatment was influenced negatively (i.e., mean gut contents were reduced) by both increased light pollution levels at each site (Fig. 3b), and light lunar phases (gut contents were least when the moon was full) (Fig. 3c). In addition, mean gut contents of invasive toads were likely to increase with increasing temperature (Fig. 3d). Rainfall and wind speed were selected in several models (Table 1), and mean gut contents of invasive toads were likely to increase slightly with increasing rainfall (Fig. 3e) and wind speed (Fig. 3f). When lights were on, percentage of flying taxa consumed by toads increased significantly (Fig. 4, p < 0.05).

**Table 1:** The result of model comparison of the generalized linear mixed models (GLMM) used to examine the relationships between gut contents of toads and various explanatory variables (experimental artificial light treatment [light-on vs light-off], ambient light pollution level, temperature, rain fall, wind speed, lunar phase, an interaction between the experimental artificial light treatment and ambient light pollution levels, an interaction between experimental artificial light treatment and lunar phase, and an interaction between present light pollution level and lunar phase).

Models No.	(Intercept)	Experimental artificial light treatment (Light-on vs Light-off)	Light pollution level	Lunar phase	Rainfall	Temperature	Wind speed	Interaction between experimental artificial light and light pollution level	Interaction between experimental artificial light and lunar phase	Interaction between light pollution level and lunar phase	df	LogLik	AIC	Delta	Weight
200	1.52	1.41	−0.84	−0.61	NA	NA	NA	−0.75	−0.69	NA	8	−63.85	143.71	0.00	0.14
224	1.50	1.69	−0.82	−0.70	0.49	0.57	NA	−0.80	−0.59	NA	10	−62.13	144.25	0.55	0.10
216	1.53	1.52	−0.84	−0.58	NA	0.34	NA	−0.77	−0.70	NA	9	−63.36	144.73	1.02	0.08
232	1.54	1.47	−0.87	−0.66	NA	NA	0.31	−0.74	−0.76	NA	9	−63.53	145.06	1.35	0.07
248	1.55	1.62	−0.87	−0.63	NA	0.40	0.37	−0.76	−0.78	NA	10	−62.60	145.20	1.49	0.06

Enclosure number was fitted as a random effect. A Gaussian distribution was identified as the error distribution. The model comparison was performed by dredge function in MuMIn package in R 3.6.0 (R Development Core Team 2019).

## Discussion

We found that the presence of artificial light in our experiment greatly increased the mass of gut contents of invasive toads, and that this effect was reduced when there was more ambient light, either from urban light pollution or natural moonlight. We also found that temperature, rainfall and wind speed had relatively weak effects on toad gut contents compared to light over the summer period of our study, and were not likely to be important drivers influencing the ecological impact of ALAN on invasive toads. These results, taken together, suggest that the food intake of invasive toads was more strongly influenced by artificial light sources than by weather effects, and that the effect of artificial light was reduced by other light sources, including natural light and ambient artificial light pollution. Moreover, flying invertabrates, such as Coleoptera, contributed strongly to the increase in gut content mass of toads in light treatments.

Lunar phase changes gradually over the synodic month, hence the impact of ALAN on invasive toads should vary cyclically corresponding to lunar phase. Although ALAN clearly provides an important food resource for invasive toads^37,38^, no one has examined the impact of lunar phase on foraging success. Lunar phase influences the attraction of biting midges (*Culicoides brevitarsis*) to artificial light, because lunar light dilutes the effect of artificial light, essentially competing with it in terms of attractiveness^6^. Our results were consistent with the possibility that bright lunar phases reduced the attraction of invertebrates to the experimental artificial light, which, in turn, lead to variation in the amount toads ate.

As with lunar illumination, we also found that the impact of ALAN was influenced by light pollution levels at each site. Our experimental light treatments were less effective at providing food for toads closer to urban centres, and thus the impact of our experimental lights, in terms of food provision, was largest in the light-naïve areas located furthest from the city. There are two plausible reasons for this effect. One is that ambient light pollution competed with the effect of our experimental artificial light, such that light pollution diluted the impact of the experimental artificial light^6^. Another reason for our observation could be that ambient light pollution, and a plethora of artificial light closer to urban centers has already degraded the invertebrate fauna at those sites. Although there is little direct experimental evidence, there is some suggestion in the literature that ambient light pollution causes declines in invertebrate populations; there are a few correlative studies reporting invertebrate population decline in association with ALAN, and species of invertebrates attracted to light have declined greatly compared to species that are not attracted to light^19,44^. Finally, moth populations in the UK and Ireland have declined as a result of ALAN^20^. In our tropical study area, there are abundant nocturnal invertebrates, and there was no indication of lower food intake in ALAN-effected areas in our study when our experimental lights were off, providing weak evidence that ALAN has not depleted invertebrate populations in our study area very much. While our study did not address the reason why ambient light pollution levels influenced the impact of our experimental lights, we suggest it was related to competition between light types in attractiveness^6^, rather than depleted invertebrate populations in our study area, although this assertion deserves further experimental examination. Possibly, invertebrates around urban areas have adapted to artificial light and have reduced flight-to-light behaviour^45^, which could also decrease numbers in urban areas.

Invasive species often inhabit environments characterised by anthropogenic disturbances, from which they may benefit, including from the presence of artificial light^26^. Invasive toads are well known to consume invertebrates attracted to artificial light around buildings, and artificial light might facilitate their invasion^38^. Our study indicated that the impact of experimental lighting on invasive toads varied depending on the light pollution level at the site. When artificial light was located in urban or peri-urban locations, the impact of experimental ALAN per light bulb seemed relatively weak. We think it most likely that this effect was due to dilution of the effect of the experimental ALAN by ambient light pollution. When the artificial light was located in peri-urban or rural areas, the impact of ALAN was relatively large. Hence, to avoid providing food resources to toads from artificial light, management of artificial light in peri-urban and rural areas, such as highway lights or scattered buildings, may be important. Additionally, light management approaches should consider the lunar cycle; light use during dark lunar phases may be most beneficial to toads, whereas the impact of light during brighter lunar phases could be relatively small.

Cage traps using ultraviolet light as a lure, which attracts invertebrates, capture toads^46^. Our results suggest that trapping may be more effective during darker lunar phases (corroborated by^47^), and in urban areas shaded from ALAN, or in peri-urban and rural areas. Considering the spatio-temporal pattern of the impact of ALAN may contribute to better management of invasive amphibians, especially if lights are used to attract insect food.

We placed all our experimental enclosures in similar habitat (open savannah woodland). Invertebrate diversity and mass may, however, vary with local environmental conditions. Future studies, should examine similarity in the invertebrate fauna among areas, and conduct this experiment at more sites, perhaps comparing different habitat types. This experiment should be repeated at other urban centres to determine if the urban effect is consistent among cities. In addition, future studies should examine the contribution of artificial light to growth rate and reproductive success of toads, to determine how ALAN influences fitness in these invasive animals.

In our experiments, the enhancement of food intake caused by experimental ALAN on invasive toads was reduced by natural light (lunar phase), and existing ambient light pollution levels. To avoid providing substantial food resources to toads from artificial lights, management of artificial light in peri-urban and rural areas, and during dark lunar phases may be advisable. On the contrary, to effectively capture toads, trapping using lights as lures at such times and places should be more successful. Considering spatio-temporal patterns in the impact of ALAN may contribute to management strategy of invasive species that benefit from it.

### Ethical statement

All procedures in this study were approved by Animal Ethics Committee at James Cook University (permit number A2520). All procedures undertaken in this study were in accordance with approved guidelines.

## Supplementary information

Supplementary information.

## Data Availability

All data analyzed in this study are included in Supplementary Table S2.
