# Supplementary material for: Impacts of artificial light on food intake in invasive toads
Source: Sci Rep. 2020 Apr 16;10:6527. doi: 10.1038/s41598-020-63503-9 (PMC7162902; doi:10.1038/s41598-020-63503-9)
Supplement: Supplementary file 1 — Supplementary information. [file 41598_2020_63503_MOESM1_ESM.pdf]

Title: Impacts of artificial light on food intake in invasive toads

Authors: Hirotaka Komine, Shinsuke Koike and Lin Schwarzkopf

Supplementary File

Supplementary Figure S1

The experimental enclosure. (a) body of enclosure. (b) a light globe before the light was turned on. (c) a light globe after the light was turned on.

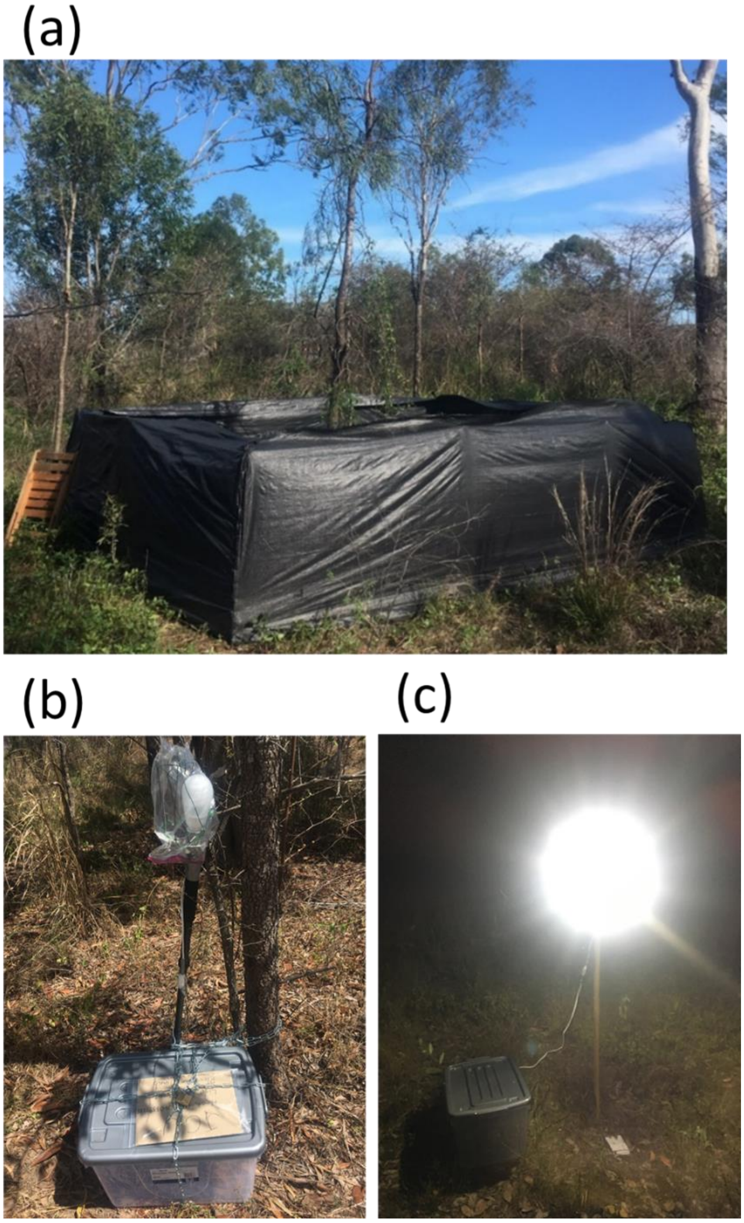

Supplementary Figure S2

The relationship between gut content mass per individual and snout-vent length.

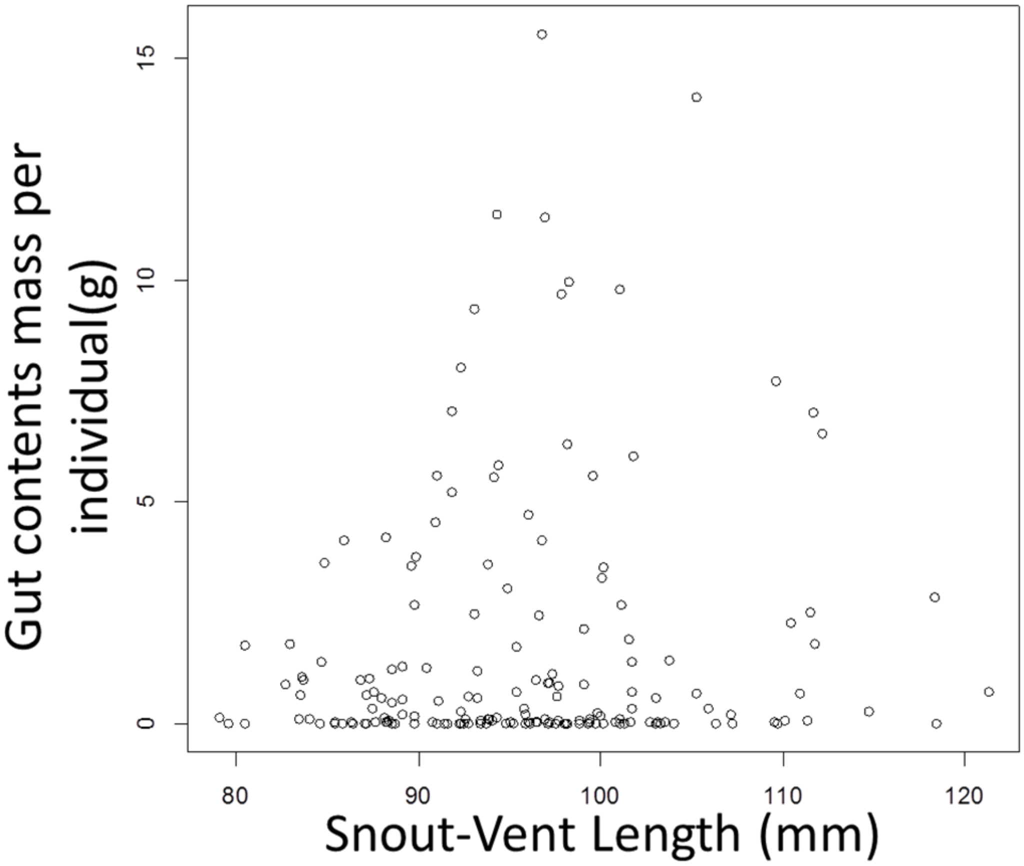

Supplementary Table S1

Result of a linear regression model testing for a relationship between gut content mass per individual and snout-vent length (SVL).

Coefficients

	Estimate	Std. Error	t value	Pr(>|t|)
(Intercept)	-2.21	2.59	-0.86	0.39
svl	0.04	0.03	1.49	0.14

Supplementary Table S2

The data that was used in the analysis.

day	month	mean SVL of toads per enclosure (mm)	site number	temperature (℃)	rainfall (mm)	wind speed (km/h)	lunar phase (%)	light pollution level (Lx)	light pollution category	light-on or light-off	mean gut contents mass per trial (g)
14	1	96.75	4	33.5	0	31	8.5	0	0	off	0.000
15	1	103.22	4	33.3	0	50	3.9	0	0	off	0.201
17	1	93.55	6	32	0	57	0	3.65	3	off	0.026
17	1	90.73	5	32	0	57	0	0.01	1	off	0.069
21	1	100.44	4	31.3	0	20	15.3	0	0	on	3.538
21	1	96.28	5	31.3	0	20	15.3	0.01	1	on	6.554
21	1	102.42	6	31.3	0	20	15.3	3.65	3	on	1.811
23	1	94.52	4	33	0	24	33	0	0	off	0.028
23	1	94.3	5	33	0	24	33	0.01	1	off	0.063
23	1	92.6	6	33	0	24	33	3.65	3	off	0.071
24	1	98.07	4	30.4	5	43	43.6	0	0	on	3.961
24	1	92.39	5	30.4	5	43	43.6	0.01	1	on	3.771
24	1	99.08	6	30.4	5	43	43.6	3.65	3	on	0.163
25	1	98.26	4	30.5	0.4	59	54.8	0	0	off	0.716
25	1	93.15	5	30.5	0.4	59	54.8	0.01	1	off	0.117
25	1	98.27	6	30.5	0.4	59	54.8	3.65	3	off	0.000
26	1	95.78	4	31.8	30.2	54	66.1	0	0	on	9.682
26	1	93.34	5	31.8	30.2	54	66.1	0.01	1	on	4.129
26	1	88.33	6	31.8	30.2	54	66.1	3.65	3	on	0.560
22	3	89.6	3	30.6	9.2	33	24.3	0.22	2	off	0.000
22	3	92.5	2	30.6	4.8	33	24.3	0.01	1	off	0.278
22	3	90.84	1	30.6	8.5	33	24.3	0	0	off	0.106
26	3	92.93	3	25.4	7.4	33	69.9	0.22	2	on	0.729
26	3	96.35	2	25.4	12.8	33	69.9	0.01	1	on	1.438

26	3	97.05	1	25.4	29	33	69.9	0	0	on	2.522
27	3	96.9	3	28.9	85.2	50	80.2	0.22	2	off	0.000
27	3	100.9	2	28.9	92	50	80.2	0.01	1	off	0.117
27	3	97.94	1	28.9	91	50	80.2	0	0	off	0.643
29	3	88.14	3	31.4	0	24	95	0.22	2	on	0.527
29	3	96.85	2	31.4	0.2	24	95	0.01	1	on	0.000
29	3	97.64	1	31.4	0	24	95	0	0	on	4.218
30	3	95.3	3	33.6	0	31	98.8	0.22	2	off	0.000
30	3	93.2	2	33.6	0	31	98.8	0.01	1	off	0.000
30	3	98.98	1	33.6	0	31	98.8	0	0	off	0.570
3	4	96	3	30.9	6.6	50	94.6	0.22	2	on	0.024
3	4	94.8	2	30.9	8.6	50	94.6	0.01	1	on	0.000
3	4	100.92	1	30.9	0	50	94.6	0	0	on	2.262
